# A new statistical workflow (R-packages based) to investigate associations between one variable of interest and the metabolome

**DOI:** 10.1007/s11306-023-02065-z

**Published:** 2023-11-30

**Authors:** Paola G. Ferrario, Achim Bub, Lara Frommherz, Ralf Krüger, Manuela J. Rist, Bernhard Watzl

**Affiliations:** 1 Department of Physiology and Biochemistry of Nutrition, Max Rubner-Institut, Haid-und-Neu-Str. 9, 76131 Karlsruhe, Germany; 2 Department of Safety and Quality of Fruit and Vegetables, Max Rubner-Institut, Haid-und-Neu-Str. 9, 76131 Karlsruhe, Germany

**Keywords:** Metabolomics, Statistical workflow, Association, Most likely transformation

## Abstract

**Introduction:**

In metabolomics, the investigation of associations between the metabolome and one trait of interest is a key research question. However, statistical analyses of such associations are often challenging. Statistical tools enabling resilient verification and clear presentation are therefore highly desired.

**Objectives:**

Our aim is to provide a contribution for statistical analysis of metabolomics data, offering a widely applicable open-source statistical workflow, which considers the intrinsic complexity of metabolomics data.

**Methods:**

We combined selected R packages tailored for all properties of heterogeneous metabolomics datasets, where metabolite parameters typically (i) are analyzed in different matrices, (ii) are measured on different analytical platforms with different precision, (iii) are analyzed by targeted as well as non-targeted methods, (iv) are scaled variously, (v) reveal heterogeneous variances, (vi) may be correlated, (vii) may have only few values or values below a detection limit, or (viii) may be incomplete.

**Results:**

The code is shared entirely and freely available. The workflow output is a table of metabolites associated with a trait of interest and a compact plot for high-quality results visualization. The workflow output and its utility are presented by applying it to two previously published datasets: one dataset from our own lab and another dataset taken from the repository MetaboLights.

**Conclusion:**

Robustness and benefits of the statistical workflow were clearly demonstrated, and everyone can directly re-use it for analysis of own data.

**Supplementary Information:**

The online version contains supplementary material available at 10.1007/s11306-023-02065-z.

## Introduction

Metabolomics has evolved as a discipline that has been widely used to measure changes in the profiles and levels of metabolites using two main distinct analytical approaches, the targeted and the nontargeted metabolite profiling. The changes in the profiles and levels of metabolites are of interest in order to understand their biological roles, in order to explore and identify biomarkers of disease, and to discover the pathogenesis of diseases. For instance, Rangel-Huerta et al. (2019) investigates the relationship between metabolites and obesity; Nguyen et al. (2021) the relationship between the metabolites and chronic hepatitis B; Kumar et al. (2020) the relationship between leptin levels and levels of metabolites of energy and hormone metabolism. However, demonstrating such relationships is often challenging. Specifically, statistical analysis is quite often one of the most challenging tasks, together with the consecutive biological interpretation of the statistical results. In fact, this task requires expertise from many disciplines and therefore interdisciplinary collaboration. In many publications, data analysis methods were reported unclearly or incompletely, so that it is impossible to follow or replicate statistical analyses, as noted by Considine et al. Considine et al. (2018). Concomitantly, in order to address the complexity of metabolomics data analysis, several tools offering help for this task have emerged. In the last decade, many research groups developed a variety of strategies and methods to extract meaningful information from metabolomics data. Just to give some examples, common web-based tools are, for instance, MetaboAnalyst (Chong et al., 2018), Workflow4Metabolomics Giacomoni et al. (2015), and Galaxy Davidson et al. (2016). Moreover, modelling data using partial least squares discriminant analysis (PLS-DA) is widespread. Mendez et al. (2020) proposed an extension of the PLS-DA to non-linear artificial neural networks, while Antonelli et al. (2019) discussed the limitations of PLS-DA when variable selection is aimed. The two reviews O’Shea and Misra (2020) and Misra (2021) summarized software resources, packages, and tools which were made available to the metabolomics research community between 2018 and 2020. The authors underline the importance of having many new tools, which represent a welcome addition from different points of view. Since only the coming years will show which software was ultimately useful and adopted in metabolomics research, the authors encourage the regular development of more software tools. In the effort to provide a contribution for statistical analysis of metabolomics data, we hereby provide a universal and widely applicable open-source statistical workflow, which is tailor-made for analysis of metabolomics data. The workflow combines many R packages, involving powerful statistical methods for data analysis. In this sense, pre-existing methods in R are re-used; concomitantly, extension and integration of further methods is easily possible. As a result, the workflow yields a table of metabolites associated with a trait of interest, together with a compact plot for high-quality results visualization. The crucial aspect of our workflow is the comprehensive consideration of the intrinsic complexity of metabolomics data, handling many aspects and characteristics of the data simultaneously. In fact, the workflow can deal with all properties of typical metabolomics datasets: the metabolites /analyte data are (i) quantified in different matrices (plasma, urine etc), (ii) measured on different analytical platforms (e.g. GC-MS, LC-MS, NMR) with different precisions, (iii) analyzed by targeted as well as non-targeted methods, (iv) scaled variously, (v) reveal heterogeneous variances, (vi) may be correlated, (vii) may have only few values (so called ties), (viii) may have values below a detection or quantification limit (left censored data), and (ix) may be incomplete (missing values). The workflow can be used for quantitative data, for semiquantitative data and also for unknowns. This means that associations can be determined for unknowns’ metabolites that have not yet been identified. Furthermore, it is important to emphasize that in most studies there are more metabolites than participants resulting in the so-called $p>N$ problem (p = number of metabolites, N = sample size). On the other hand, it should be considered that the variable of interest can be modelled quantitatively as well as qualitatively, and that each metabolite association can be calculated separately or together with other analytes as metabolite patterns (univariate vs. multivariate analysis). Finally, the role of other important participant attributes, such as sex, BMI (i.e. competing covariates) needs to be considered as well. Our workflow addresses all these specific issues by appropriate statistical analysis of metabolomics data. In this paper, we first explain the principle steps of the proposed statistical approach. Secondly, workflow utility and output are demonstrated by applying it to two previously published and well-documented datasets: one dataset from our own lab and another dataset taken from the repository MetaboLights Haug et al. (2012). These exemplary re-evaluations shall support clarity and transparency in metabolomics data analysis, as suggested by Considine et al. (2018). Specifically, we re-analyzed two human studies concerning metabolites: a cross-sectional study with the aim to characterize the metabolome of healthy individuals, and another study with the aim to investigate the impact of age on the urinary metabolome.

## Methods

### Selected studies for re-evaluation using our workflow

#### The cross-sectional study Karlsruhe Metabolomics and Nutrition (KarMeN)

The first dataset used came from the study presented and described in detail by Bub et al. (2016), the KarMeN study. This was a cross-sectional study, conducted in Germany between 2011 and 2013. Briefly, 301 healthy adults, 172 men, 129 women, 18–80 years old were recruited. They were subjected to a standardized examination schedule for a characterization by anthropometric, functional, and clinical parameters. Moreover, urine and blood samples were collected for metabolomics analysis with different analytical methods. This yielded in total more than 400 analytes in plasma and over 500 analytes in urine. Originally, predictive modelling was applied on these data using different machine learning algorithms to investigate associations of metabolites with age and sex Rist et al. (2017).

#### The analysis of the human adult urinary metabolome variations with age by Thévenot et al. (2015)

The second dataset we re-analyzed came from the study presented by Thévenot et al. (2015), which had the aim to investigate the impact of age on the urinary metabolome. Urine samples were collected from a cohort of 183 human adults during their routine examination. Thevenot et al. started with 258 metabolites measured by LC-HRMS in total and, after some quality control operations, finally considered 170 metabolites for evaluation. Of these 170 metabolites, 120 are available online. Specifically, the online repository ( compare the data availability statement) contains the data set from the negative ionization mode while the associated publication also describes data from the positive ionization mode.

The data were modelled by orthogonal partial least-squares. The effects for the association between the metabolites and age were given by $\log 2$ ratios. Specifically, metabolites were sorted by decreasing $\log 2$ ratios of the predicted intensities by the model. The statistical analyses were conducted as univariate analysis of the association of age with the 120 metabolites, respectively.

### Statistical analysis as realized in our workflow

In order to detect the relationship between a continuous variable *X* (e.g., age) on a total number of *p* human metabolites $Y_1, Y_2, \dots , Y_p,$ (i.e., i metabolites with i from 1 to p) the association between the covariate of interest *X* and the *p* metabolites was expressed by the following regression models:

$$\begin{aligned} \left\{ \begin{aligned} h^{[a]}_1(Y_1) =&\beta _1^{[a]} X+ \epsilon _1^{[a]} \\ \vdots \\ h^{[a]}_p(Y_p) =&\beta _p^{[a]} X+ \epsilon _p^{[a]}, \end{aligned} \right. \qquad \text {or}~ \left\{ \begin{aligned} h^{[l]}_1(Y_1) =&\beta _1^{[l]} \log X+ \epsilon _1^{[l]} \\ \vdots \\ h^{[l]}_p(Y_p) =&\beta _p^{[l]} \log X+ \epsilon _p^{[l]}, \end{aligned} \right. \end{aligned}$$

Here, $h_i$ were various, strictly monotonically increasing transformations of the metabolites $Y_i$ (as explained below). $\beta _i$ were the regression parameters, whose interpretation resulted from the a priori chosen error distribution of the errors $\epsilon _i$.

In order to be receptive for different association patterns between the metabolites $Y_i$ and the variable of interest *X*, the variable of interest *X* was taken unmodified as well as modified by the logarithm, i.e., considering $\log (X)$ (Tukey et al., 1985). Specifically, this means that we had two association models, one with covariate *X*, the other with its logarithm $\log (X)$. Consequently, there were a total of two regression models per each metabolite $Y_i$, expressed respectively by the notation [a] and [l], where [l] stands for the logarithm (for positive *X*). The R function tukeytrend::dosescalett allows to consider and store *X* and its logarithm simultaneously, where the different operations on *X* (like the calculation of the logarithm) constitute the so-called "metameters".

In order to be open for different shapes of the conditional distribution function of Y, we considered so-called transformation models. The core idea of any transformation model is the application of a transformation function *h* for the reformulation of the unknown distribution function $P(Y<y)$ as $P(h(Y)<= h(y))$. Specifically, the functions $h_i$ were estimated from the data by the so-called most likely transformation (Hothorn et al., 2018): This estimation was embedded in a maximum likelihood framework and it was facilitated by parametrization of the function *h*. Specifically, the parametrization was conducted by Bernstein polynomials. In this way, the conditional distribution of a metabolite $Y_i$ given the variable of interest *X* was estimated from a flexible parametric model, with the help of the R function mlt::mlt or of the function tram::boxCox.

Moreover, the metabolites affected by a limit of quantification were considered as left censored variables. The R function survival::Surv was involved for calculation with censored variables. This function stored the variables together with the information if these were censored or not as well as the corresponding censorship value. Finally, the metabolites affected by many repeated values were considered as having so-called ties. This is the case when many observations take the same value. In many cases ties resulted from insufficient precision, in other cases ties were caused by the discrete character of a variable. The way we chose to "break" ties was to regard them as censored, interval variables. Again, the R function survival::Surv was involved for calculation for variables with ties, considering these as interval variables.

The null hypothesis was that there is no association between the metabolites $Y_i$ and the variable of interest *X* or between the metabolites $Y_i$ and the logarithm of the variable of interest, $\log (X)$. The alternative hypothesis was that at least one model shows association, that is:

$$\begin{aligned} H_0: \beta _i^{[a]}=\beta _i^{[l]}=0 \quad \text {vs.}\quad H_1: \beta _i^{[a]}\ne 0 \text { or } \beta _i^{[l]} \ne 0 \end{aligned}$$

**Fig. 1:**
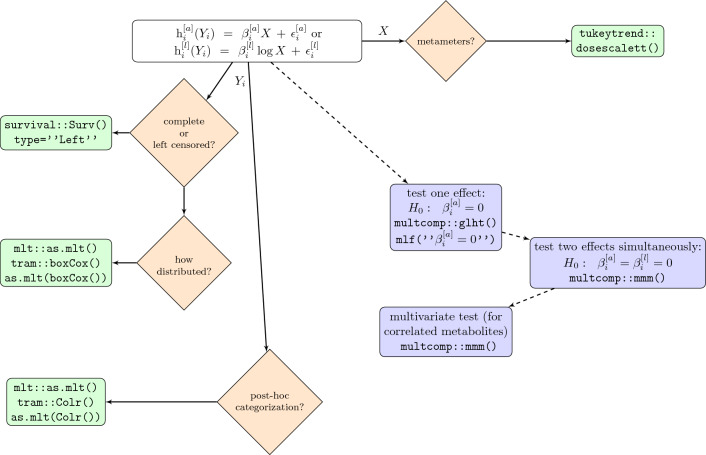
Visualization of the workflow. Orange diamonds represents mandatory questions about data characteristics or analysis strategy. Green rectangles list the R packages and functions involved as proposed solution. Blue rectangles between dashed lines indicate possible inference steps

The equivalent multivariate hypothesis generalized this for all metabolites: Is there an association between all the metabolites $Y_i$, $i=1...p$ and the variable of interest *X*? For the in total $2*p$ models, the correlation between the marginal test statistics was calculated by so-called multiple marginal models (R function multcomp::mmm) and taken into account for the joint inference by general linear hypothesis (R function multcomp::glht) (Pipper et al., 2012). This corresponds to a multivariate analysis, correcting for multiplicity. The full-page-figure (Fig. 1) schematically displays the main principles behind the workflow, the R packages and functions involved, indicating possible inference steps.

## Results

In this section, we demonstrate the principle of our workflow by presenting the results of the statistical analyses applied to the two representative studies, the KarMeN study and the study by Thevenot et al.

### The KarMeN cross-sectional study as explanatory data example

In order to demonstrate the workflow step-by-step, we used an exemplary dataset consisting of two (continuous) metabolites from the KarMeN Study (Bub et al., 2016) and (Frommherz et al., 2016) for the sake of explanation; the code can be found in the supplementary information. Specifically, the chosen metabolites were: the plasma metabolite GUDCA, i.e., glycoursodeoxycholic acid, a bile acid quantified by LC-MS; and the plasma metabolite C10:0, decanoic acid, a saturated fatty acid quantified by GC-MS. This provided a particular case example, since GUDCA was affected by a limit of quantification (LOQ) of 25 nM (left censored) and C10:0 was affected by many repeated values (ties). Specifically, 193 individuals had the same value for C10:0 equal to 0.46. The variable of interest was the age. The aim was to investigate associations between age and the metabolites GUDCA and C10:0, respectively and jointly.

The results of this calculation is a table containing the estimated effects and the simultaneous confidence intervals for arithmetic as well as logarithmic dependencies (Table 1). The workflow reveals a strong association between GUDCA and age, and no association between C10:0 and age. This corresponds to the results for these two analytes already documented in Rist et al. (2017). Also after adjustment for BMI, the association between GUDCA and age is confirmed as well as the fact that there is no association between the metabolite C10:0 and age.

**Table 1:** Effects and simultaneous confidence intervals for the association between the metabolite GUDCA and age and between the metabolite C10:0 and age, considering age unmodified (i.e., metameter "arithmetic") and the logarithm of age (metameter "logarithmic")

Metabolite	Metameter	Effect	Lower limit	Upper limit
GUDCA	Arithmetic	− 0.016	− 0.025	− 0.008
GUDCA	Logarithmic	− 0.738	− 1.094	− 0.381
C10:0	Arithmetic	0.003	− 0.005	0.012
C10:0	Logarithmic	0.124	− 0.225	0.474

### The Thevenot et al. study as reproducible study example

**Fig. 2:**
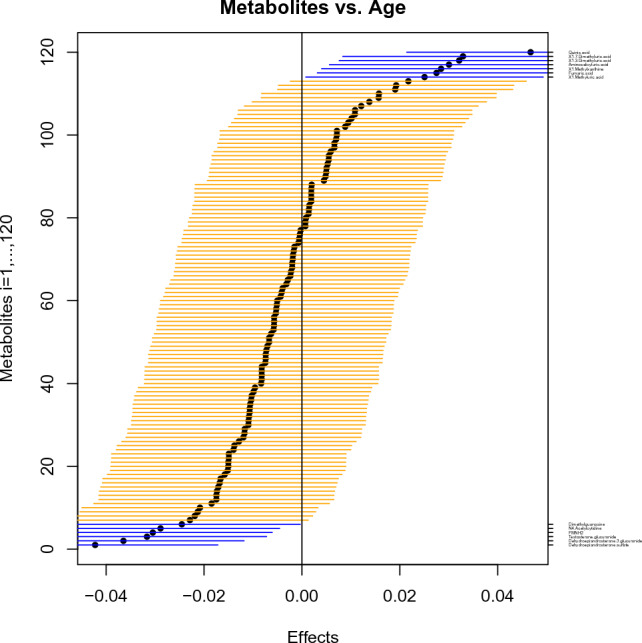
Impact of age on 120 urine metabolites for 183 adults under a linear model, visualized by simultaneous confidence intervals, ordered by increasing effects, given as horizontal lines around black circles. The blue lines are the metabolites that show association with age: Quinic acid, 1.7-Dimethyluric acid, 1.3-Dimethyluric acid, Aminosalicyluric acid, 1-Methylxanthine, Fumaric acid, and 1-Methyluric acid (positively associated); Dimethylguanosine, N4-Acetylcytidine, FMNH2, Testosterone glucuronide, Dehydroepiandrosterone 3- glucuronide, Dehydroepiandrosterone sulfate (negatively associated)

**Fig. 3:**
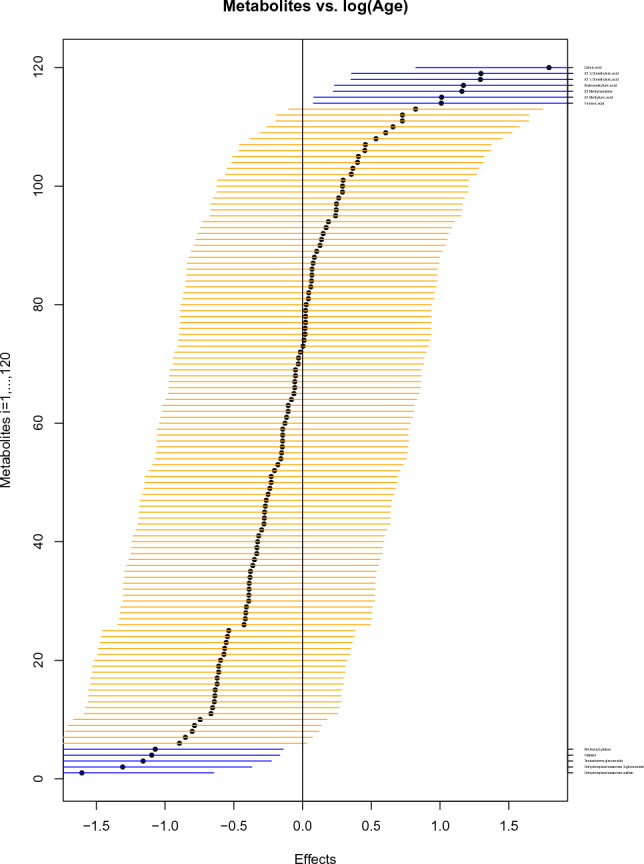
Impact of age on 120 urine metabolites for 183 adults under a logarithmic model, visualized by simultaneous confidence intervals, ordered by increasing effects, given as horizontal lines around black circles. The blue lines are the metabolites that show association with $\log$(age): Quinic acid, 1.3-Dimethyluric acid, 1.7-Dimethyluric acid, Aminosalicyluric acid, 1-Methylxanthine, 1-Methyluric acid, and Fumaric acid (positively associated); N4-Acetylcytidine, FMNH2, Testosterone glucuronide, Dehydroepiandrosterone 3- glucuronide, Dehydroepiandrosterone sulfate (negatively associated)

In order to further illustrate how our workflow works, we re-analyzed data by Thévenot et al. (2015), investigating the association of age with the urinary metabolome (compare the section Methods); the code can be found in the supplementary information. For this purpose, the association of 120 metabolites with the age of 183 adults were investigated. Specifically, the associations of age were considered simultaneously for all metabolites. The complete results of applying the workflow to the data was visualized with one single plot: Fig. 2 displays one confidence interval for each metabolite; together, the simultaneous confidence intervals for all 120 metabolites are ordered by increasing effects for linear association with age, allowing scale-independent interpretation. The confidence intervals not transgressing the vertical line (for effect=0) describe the metabolites associated with age; specifically, they show a positive association when they are at the right-hand side of the vertical line, and a negative association when they are on the left-hand side. The calculated effects are listed in table-form, together with the low and the upper value of the confidence intervals (compare the supplementary material). In the same way, simultaneous confidence intervals for logarithmic associations between metabolites and age are displayed (Fig. 3). The results obtained by Thévenot et al. (2015) and by our workflow were summarized in a list and compared (Table 2): the 52 metabolites reported in Thevenot et al. (2015) as associated with age are sorted by decreasing log2 ratios as in the original work. The metabolites identified by our workflow as significantly associated with age are in bold and blue. These are less than the metabolites identified Thévenot et al. (2015). In fact, Thévenot et al. (2015) conducted a univariate analysis with significance threshold given by a p-value equal to 0.05, while our workflow conducted a multivariate analysis, constructing simultaneous confidence intervals and therefore considering also the number of involved metabolites. This is appropriate when dealing with many metabolites at the same time. As a consequence, the probability for false-positive results is lower.

**Table 2:** List of the 52 significant metabolites identified in Thévenot et al. (2015), sorted by decreasing $\log 2$ ratios for association with age. In italic, the metabolites that are not available. In bold, the metabolites identified by the workflow. For these, the $\log 2$ ratios calculated by Thévenot et al. (2015) are also reported

Metabolites	log2 ratio of age in Thévenot et al. (2015)
*Aniline isomer*	
*Indoleacetic acid isomer*	
*Pyridoxic acid isomer 2*	
*Nicotinic acid isomer*	
*Caffeine*	
**Quinic acid**	4.4
**Aminosalicyluric acid**	3.6
*Adipoylcarnitine*	
**1,7-Dimethyluric acid**	3.3
**1,3-Dimethyluric acid**	3.1
*Paraxanthine/Theophylline*	
**1-Methylxanthine**	1.9
**Fumaric acid**	1.8
Pyrroledicarboxylic acid	
**1-Methyluric acid**	1.6
*Hydroxyanthranilic acid isomer*	
2-acetamido-4-methylphenyl acetate	
Acetylphenylalanine	
Methoxysalicylic acid isomer	
Tetrahydrohippuric acid	
Normetanephrine isomer	
2-Hydroxybenzyl alcohol	
Threo-3-Phenylserine	
N-Acetyltryptophan	
Deoxyhexose	
Pentose	
**Dehydroepiandrosterone sulfate**	− 7
**Testosterone glucuronide**	− 3.1
**Dehydroepiandrosterone 3-glucuronide**	− 2.9
**FMNH2**	− 2.6
**N4-Acetylcytidine**	− 2.3
6-(carboxymethoxy)-hexanoic acid	
*Tryptamine*	
4-Hydroxybenzoic acid	
Methylinosine	
*Indoleacetyl glutamine*	
*Decanoylcarnitine isomer*	
*Decenoylcarnitine isomer 2*	
Tryptophan	
**Dimethylguanosine**	$-$0.91
Hydroxysuberic acid isomer 2	
Aspartic acid	
Pyridylacetylglycine	
N-Acetyl-aspartic acid	
Kynurenic acid	
Heptylmalonic acid	
4-Acetamidobutanoic acid isomer 2	
N-Acetyltryptophan isomer 3	
3-Methylcrotonylglycine	
(gamma)Glu-Leu/Ile	
Pantothenic acid	
5-Hydroxyindoleacetic acid	

## Discussion and conclusion

In the present article, we propose a statistical workflow based on a combination of several R packages (survival, tram, mlt, multcomp), considering common issues with metabolomics data, such as the fact that metabolomics data are scaled differently, are platform-dependent, are heterogeneous, and are sometimes not detectable below a certain limit. Moreover, by considering the most likely transformation function for each metabolite, we enable the metabolites to be differently distributed, left censored and to have different patterns of association with the variable of interest, or with its logarithm as well. According to current statistical approaches, we have considered the correlation between metabolites in order to adjust for multiplicity in a less conservative way than with classical approaches (i.e., the Bonferroni-correction).

More specifically, the strengths of the proposed workflow are the following:

There are no a priori assumptions about the distribution of $Y_i$, since it is unrealistic to assume the same error distribution for each of the p metabolites. Instead, a metabolite-specific data-driven transformation function is considered. mlt provides a comparable analysis of such differently scaled metabolites and therefore enables scale-independent interpretation of the results, which is particularly helpful when searching for associations between one specific variable of interest and multiple metabolites simultaneously.

The workflow considers the fact that the p metabolites are a mixture of completely measured metabolites, but also left censored metabolites (with values below the limit of detection or quantification), which is an intrinsic property of the chemical measurement of metabolites. Moreover, some metabolites have many ties and these are also integrated in analysis. Whether a feature can be attributed to a certain molecule or not, is not relevant for the workflow. Therefore, a description consisting of a pair of m/z and RT values is certainly compatible with the workflow.

Different association patterns between the metabolites $Y_i$ and the variable of interest X are “allowed”: The workflow is also able to detect non-linear associations. Each metabolite gets its own “suitable” model for association with the variable of interest as result of a maximization process without need for explicit formulation of some parameters.

The adjustment for multiple comparisons considers another intrinsic property of the metabolites, namely that they are often correlated in subgroups. This information has been included and leads to adjustments for multiplicity that can be less conservative compared to approaches that ignore it.

A possible limitation of the workflow occurs if there is a large proportion of values below a detection limit. In that case, it could be difficult to identify the Bernstein polynomials and hence to calculate the most likely transformation.

There are also possible extensions of the workflow, which generalize the proposed procedures. For instance, a possible extension is represented by the so-called continuous outcome logistic regression, a technique that has recently been proposed by Lohse et al. (2017). The continuous outcome logistic regression can be applied in case there is interest in categorization of the metabolites. This procedure represents a valid alternative to post-hoc categorizations. Specifically, this type of regression makes it possible to consider the association between the variable of interest and the metabolites by odds ratios that can be evaluated for all potential values or cut-offs of the metabolites. Therefore, the step by the continuous outcome logistic regression is also included in Fig. 1. According to the recommendations of Open Science Spellman et al. (2018), we are sharing the code in its entirety and we have demonstrated the workflow using publicly available data. The shared code can be re-used on the proposed data and on other datasets to achieve progress in metabolomics research.

### Supplementary Information

Below is the link to the electronic supplementary material.

Supplementary file 1 (XLSX 30 KB)

Supplementary file 2 (R 3 KB)

Supplementary file 3 (RNW 9 KB)

Supplementary file 4 (PDF 150 KB)

## Data Availability

The datasets analysed during the current study are available in the online data repository MetaboLights, with ID MTBLS404. Further data used for demonstration (from the KarMeN study) are available in the additional files. The whole code that demonstrates the workflow is available in the additional files too.
