# Supplementary material for: A new statistical workflow (R-packages based) to investigate associations between one variable of interest and the metabolome
Source: Metabolomics. 2023 Nov 30;20(1):2. doi: 10.1007/s11306-023-02065-z (PMC10689553; doi:10.1007/s11306-023-02065-z)
Supplement: Supplementary file 4 — (PDF 150 KB) [file 11306_2023_2065_MOESM4_ESM.pdf]

Additional file

Application of the workflow to a real, available dataset: Urinary metabolome variation with age

Contents

1 Aim **1**

2 Data **1**

3 A little descriptive statistics **1**

4 Re-analysation of data **3**

1 Aim

The aim of this report is to re-analyse large-scale data that are available in literature using the presented workflow. Thus, we demonstrate how the workflow works in practice, which assumptions are required and how the results can be represented in form of, for instance, confidence intervals, plots, lists of p-values, etc....The code is completely reproducible.
To this end, we considered a cohort consisting of 120 analytes measured by LC-HRMS for 183 adults (compare the section below). In particular, we focussed on the association between the analytes and age as continuous covariate of interest.

2 Data

Consider the article:
Thévenot et al. (2015) Analysis of the Human Adult Urinary Metabolome Variations with age, Body Mass Index, and Gender by Implementing a Comprehensive Workflow for Univariate and OPLS Statistical Analyses. *J Proteome Res*, **14(8)**, 3322-3335.
DOI: `10.1021/acs.jproteome.5b00354`
`https://pubs.acs.org/doi/10.1021/acs.jproteome.5b00354` and the data available together with the article

- `sacurine-neg_sampleMetadata.tsv`, downloaded from `https://workflow4metabolomics.org/lcms_dataset-sacurine` (March 09th, 2023)
- `m_sacurine_v2_maf.tsv`, downloaded from `https://www.ebi.ac.uk/metabolights/MTBLS404/files` (March 09th, 2023)

PLEASE DOWNLOAD THESE FILES AND SAVE THEM IN THE SAME DIRECTORY AS THIS ".RNW" FILE

3 A little descriptive statistics

Draw gender-specific age and BMI distributions

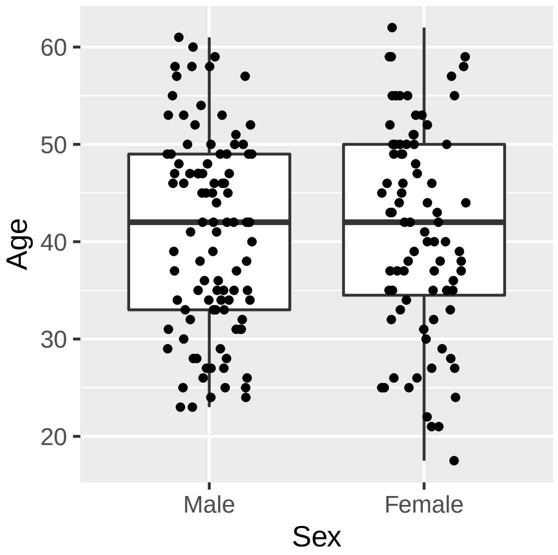

Figure 1: Gender-specific age distribution.

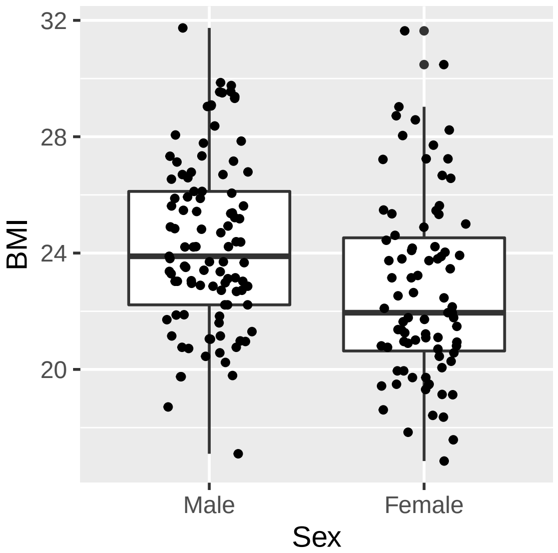

Figure 2: Gender-specific BMI distribution.

4 Re-analysation of data

Is there any association between each metabolite and age or between each metabolite and log(age), respectively?
Run for this the workflow on the data

```
analyt <- make.names(analyt_orig <- colnames(data.thevenot)[-(1:4)])
colnames(data.thevenot)[-(1:4)] <- analyt

dd <- dosescalett(data = data.thevenot, dose = "age",
                  scaling = c("ari","log"))$data

make_str <- function(a) {
  cm_ari <- as.mlt(BoxCox(as.formula(paste(a, " ~ ageari")), data = dd))
  cm_log <- as.mlt(BoxCox(as.formula(paste(a, " ~ agelog")), data = dd))
  cm_mmm <- glht(mmm(ari = cm_ari, log = cm_log),
                 mlf(ari = "ageari=0", log = "agelog=0"))
  list(cf = confint(cm_mmm)$confint,
       pvals = summary(cm_mmm)$test$pvalue,
       logLik = c(ageari = logLik(cm_ari),
                  agelog = logLik(cm_log)))
}
res <- lapply(analyt, make_str)
```

Analyte	Age	Effect	Confint	p-Value	logLik
Quinic acid	ari	0.05	(0.03– 0.06)	<0.001	-3055.40
	log	1.79	(1.20– 2.38)	<0.001	-3055.31
Dehydroepiandrosterone sulfate	ari	-0.04	(-0.06—0.03)	<0.001	-3130.04
	log	-1.60	(-2.19—1.02)	<0.001	-3130.23
Dehydroepiandrosterone 3-glucuronide	ari	-0.04	(-0.05—0.02)	<0.001	-2473.95
	log	-1.31	(-1.88—0.74)	<0.001	-2475.43
1,3-Dimethyluric acid	ari	0.03	(0.02– 0.05)	<0.001	-2978.91
	log	1.30	(0.73– 1.87)	<0.001	-2977.89
1,7-Dimethyluric acid	ari	0.03	(0.02– 0.05)	<0.001	-3162.10
	log	1.29	(0.72– 1.87)	<0.001	-3161.58
Testosterone glucuronide	ari	-0.03	(-0.05—0.02)	<0.001	-2426.60
	log	-1.16	(-1.73—0.59)	<0.001	-2427.53
Aminosalicyluric acid	ari	0.03	(0.02– 0.04)	<0.001	-2735.07
	log	1.17	(0.60– 1.74)	<0.001	-2734.57
FMNH2	ari	-0.03	(-0.05—0.02)	<0.001	-2049.05
	log	-1.10	(-1.66—0.53)	<0.001	-2050.00
1-Methylxanthine	ari	0.03	(0.01– 0.04)	<0.001	-3176.16
	log	1.16	(0.59– 1.73)	<0.001	-3175.46
N4-Acetylcytidine	ari	-0.03	(-0.04—0.01)	<0.001	-2515.49
	log	-1.07	(-1.64—0.51)	<0.001	-2515.94
Fumaric acid	ari	0.03	(0.01– 0.04)	<0.001	-2515.98
	log	1.01	(0.44– 1.57)	<0.001	-2516.51
1-Methyluric acid	ari	0.03	(0.01– 0.04)	<0.001	-3241.22
	log	1.01	(0.45– 1.57)	<0.001	-3240.60

Dimethylguanosine	ari	-0.02	(-0.04—0.01)	<0.001	-2652.98
	log	-0.90	(-1.46—0.33)	0.0016	-2653.48
6-(carboxymethoxy)-hexanoic acid	ari	-0.02	(-0.04—0.01)	0.0020	-2662.46
	log	-0.85	(-1.41—0.29)	0.0026	-2662.90
Methylinosine	ari	-0.02	(-0.04—0.01)	0.0030	-2308.39
	log	-0.79	(-1.34—0.23)	0.0054	-2308.92
Pyrroledicarboxylic acid	ari	0.02	(0.01– 0.04)	0.0032	-2496.21
	log	0.82	(0.26– 1.38)	0.0036	-2496.33
N-Acetyltryptophan isomer 3	ari	-0.02	(-0.04—0.01)	0.0040	-2721.70
	log	-0.80	(-1.36—0.24)	0.0045	-2721.85
Aspartic acid	ari	-0.02	(-0.04—0.01)	0.0049	-2741.63
	log	-0.74	(-1.30—0.18)	0.0086	-2742.15
Pentose	ari	0.02	(0.00– 0.03)	0.0089	-3131.41
	log	0.73	(0.17– 1.28)	0.0100	-3131.51
Acetylphenylalanine	ari	0.02	(0.00– 0.03)	0.0096	-2616.93
	log	0.73	(0.17– 1.28)	0.0100	-2616.92
4-Hydroxybenzoic acid	ari	-0.02	(-0.03—0.00)	0.012	-2419.62
	log	-0.67	(-1.22—0.11)	0.018	-2419.83
gamma-Glu-Leu	ari	-0.02	(-0.03—0.00)	0.018	-2553.13
	log	-0.62	(-1.18—0.07)	0.028	-2553.42
gamma-Glu-Ile	ari	-0.02	(-0.03—0.00)	0.018	-2553.13
	log	-0.62	(-1.18—0.07)	0.028	-2553.42
3-Methylcrotonylglycine	ari	-0.02	(-0.03—0.00)	0.019	-2903.84
	log	-0.64	(-1.20—0.08)	0.023	-2904.03
N-Acetyltryptophan	ari	0.02	(0.00– 0.03)	0.034	-2334.38
	log	0.66	(0.10– 1.21)	0.020	-2333.92
Pantothenic acid	ari	-0.02	(-0.03—0.00)	0.025	-2903.71
	log	-0.65	(-1.21—0.10)	0.021	-2903.53
Pyridoxic acid isomer 1	ari	-0.02	(-0.03—0.00)	0.021	-3094.71
	log	-0.63	(-1.19—0.08)	0.025	-3094.70
N-Acetyl-aspartic acid	ari	-0.02	(-0.03—0.00)	0.023	-2437.82
	log	-0.64	(-1.19—0.08)	0.024	-2437.88
Threonic acid	ari	-0.01	(-0.03—0.00)	0.043	-3182.11
	log	-0.61	(-1.17—0.05)	0.031	-3181.78
Erythronic acid	ari	-0.01	(-0.03—0.00)	0.043	-3182.11
	log	-0.61	(-1.17—0.05)	0.031	-3181.78
Methoxysalicylic acid isomer	ari	0.02	(0.00– 0.03)	0.033	-2524.35
	log	0.60	(0.05– 1.16)	0.033	-2524.09
Pyridylacetylglycine	ari	-0.02	(-0.03—0.00)	0.033	-2744.36
	log	-0.60	(-1.15—0.04)	0.035	-2744.41
Heptylmalonic acid	ari	-0.02	(-0.03—0.00)	0.04	-2932.10
	log	-0.56	(-1.11—0.00)	0.05	-2932.28
5-Hydroxyindoleacetic acid	ari	-0.02	(-0.03—0.00)	0.043	-2411.83
	log	-0.54	(-1.09– 0.02)	0.059	-2412.11
Hydroxysuberic acid isomer 2	ari	-0.02	(-0.03—0.00)	0.043	-2460.11
	log	-0.57	(-1.13—0.01)	0.044	-2460.30
Hydroxysuberic acid isomer 1	ari	-0.01	(-0.03– 0.00)	0.063	-2483.96
	log	-0.57	(-1.12—0.01)	0.045	-2483.68
o-Hydroxyphenylacetic acid	ari	-0.01	(-0.03– 0.00)	0.060	-2712.69
	log	-0.55	(-1.10– 0.01)	0.054	-2712.60

Threo-3-Phenylserine	ari	0.01	(-0.00– 0.03)	0.063	-2922.50
	log	0.53	(-0.02– 1.09)	0.059	-2922.43
Glycocholic acid isomer 1	ari	-0.01	(-0.03– 0.00)	0.082	-1985.71
	log	-0.41	(-0.96– 0.14)	0.151	-1986.18
Deoxyhexose	ari	0.01	(-0.00– 0.03)	0.10	-2558.72
	log	0.46	(-0.10– 1.01)	0.11	-2558.76
Tryptophan	ari	-0.01	(-0.03– 0.00)	0.11	-2528.43
	log	-0.42	(-0.97– 0.14)	0.15	-2528.66
2-Hydroxybenzyl alcohol	ari	0.01	(-0.00– 0.03)	0.15	-2356.93
	log	0.45	(-0.10– 1.01)	0.11	-2356.77
3,7-Dimethyluric acid	ari	-0.01	(-0.03– 0.00)	0.11	-2715.96
	log	-0.39	(-0.94– 0.16)	0.17	-2716.33
Kynurenic acid	ari	-0.01	(-0.03– 0.00)	0.12	-2934.42
	log	-0.41	(-0.96– 0.14)	0.15	-2934.61
2-Aminoadipic acid	ari	-0.01	(-0.03– 0.00)	0.16	-2336.25
	log	-0.43	(-0.98– 0.13)	0.14	-2336.19
2,2-Dimethylglutaric acid	ari	-0.01	(-0.03– 0.00)	0.14	-2581.16
	log	-0.38	(-0.93– 0.17)	0.18	-2581.33
p-Hydroxymandelic acid	ari	-0.01	(-0.03– 0.00)	0.14	-2764.68
	log	-0.39	(-0.94– 0.17)	0.18	-2764.83
Hydroxyphenyllactic acid	ari	0.01	(-0.00– 0.03)	0.15	-3192.65
	log	0.40	(-0.15– 0.95)	0.16	-3192.79
Acetaminophen glucuronide	ari	-0.01	(-0.03– 0.00)	0.15	-2628.29
	log	-0.10	(-0.66– 0.45)	0.76	-2628.34
Oxoglutaric acid	ari	-0.01	(-0.03– 0.00)	0.15	-3027.88
	log	-0.35	(-0.90– 0.20)	0.22	-3028.10
N2-Acetylaminoadipic acid	ari	-0.01	(-0.03– 0.00)	0.15	-2686.55
	log	-0.39	(-0.94– 0.16)	0.17	-2686.61
5-Sulfosalicylic acid	ari	0.01	(-0.00– 0.02)	0.20	-2807.74
	log	0.41	(-0.15– 0.96)	0.15	-2807.52
Asp-Leu/Ile isomer 2	ari	-0.01	(-0.02– 0.00)	0.16	-2467.47
	log	-0.33	(-0.88– 0.22)	0.24	-2467.78
3-Indole carboxylic acid glucuronide	ari	-0.01	(-0.02– 0.00)	0.17	-2842.79
	log	-0.38	(-0.93– 0.17)	0.18	-2842.87
Valerylglycine isomer 1	ari	-0.01	(-0.02– 0.00)	0.19	-2638.99
	log	-0.39	(-0.94– 0.16)	0.17	-2638.94
Benzoic acid isomer	ari	0.01	(-0.00– 0.02)	0.18	-2682.48
	log	0.37	(-0.19– 0.92)	0.20	-2682.61
4-Acetamidobutanoic acid isomer 2	ari	-0.01	(-0.02– 0.00)	0.20	-3120.34
	log	-0.36	(-0.91– 0.19)	0.21	-3120.37
Hippuric acid	ari	0.01	(-0.01– 0.02)	0.24	-3575.89
	log	0.35	(-0.20– 0.91)	0.22	-3575.82
Gluconic acid and/or isomers	ari	-0.01	(-0.02– 0.01)	0.28	-2932.97
	log	-0.33	(-0.88– 0.22)	0.25	-2932.89
4-Acetamidobutanoic acid isomer 3	ari	-0.01	(-0.02– 0.01)	0.28	-2867.23
	log	-0.33	(-0.88– 0.23)	0.26	-2867.18
Glu-Val	ari	-0.01	(-0.02– 0.01)	0.27	-2715.28
	log	-0.32	(-0.87– 0.23)	0.27	-2715.28
Sebacic acid	ari	-0.01	(-0.02– 0.01)	0.27	-2940.18
	log	-0.26	(-0.81– 0.29)	0.36	-2940.37

Glycocholic acid isomer 3	ari	-0.01	(-0.02– 0.01)	0.28	-2161.06
	log	-0.24	(-0.79– 0.31)	0.42	-2161.33
p-Anisic acid	ari	-0.01	(-0.02– 0.01)	0.38	-2865.82
	log	-0.30	(-0.85– 0.25)	0.30	-2865.69
Phe-Tyr-Asp (and isomers)	ari	0.01	(-0.01– 0.02)	0.35	-2884.36
	log	0.30	(-0.26– 0.85)	0.31	-2884.28
4-Methylhippuric acid	ari	0.01	(-0.01– 0.02)	0.38	-2729.32
	log	0.29	(-0.26– 0.84)	0.31	-2729.11
3-Methylhippuric acid	ari	0.01	(-0.01– 0.02)	0.38	-2729.32
	log	0.29	(-0.26– 0.84)	0.31	-2729.11
Valerylglycine isomer 2	ari	-0.01	(-0.02– 0.01)	0.32	-2899.58
	log	-0.28	(-0.83– 0.28)	0.34	-2899.62
Porphobilinogen	ari	-0.01	(-0.02– 0.01)	0.33	-2807.25
	log	-0.28	(-0.83– 0.27)	0.33	-2807.26
Pyruvic acid	ari	-0.01	(-0.02– 0.01)	0.33	-2785.91
	log	-0.27	(-0.82– 0.28)	0.35	-2785.95
Isovalerylalanine isomer	ari	-0.01	(-0.02– 0.01)	0.34	-2459.47
	log	-0.20	(-0.75– 0.35)	0.49	-2459.63
Glyceric acid	ari	-0.01	(-0.02– 0.01)	0.42	-2630.90
	log	-0.28	(-0.83– 0.28)	0.34	-2630.73
Pyrocatechol sulfate	ari	0.01	(-0.01– 0.02)	0.35	-3409.57
	log	0.26	(-0.29– 0.82)	0.37	-3409.61
N-Acetylisoleucine	ari	-0.01	(-0.02– 0.01)	0.35	-2741.28
	log	-0.25	(-0.80– 0.30)	0.39	-2741.36
2-acetamido-4-methylphenyl acetate	ari	0.01	(-0.01– 0.02)	0.36	-2387.78
	log	0.25	(-0.31– 0.80)	0.40	-2387.86
Mevalonic acid isomer 1	ari	-0.01	(-0.02– 0.01)	0.38	-2426.41
	log	0.01	(-0.54– 0.57)	0.99	-2426.91
m-Hydroxyhippuric acid	ari	0.00	(-0.01– 0.02)	0.52	-3009.34
	log	0.25	(-0.31– 0.80)	0.40	-3009.18
Citric acid	ari	0.01	(-0.01– 0.02)	0.48	-3660.75
	log	0.24	(-0.31– 0.79)	0.41	-3660.63
3,5-dihydroxybenzoic acid	ari	-0.01	(-0.02– 0.01)	0.46	-2572.62
	log	-0.23	(-0.78– 0.32)	0.44	-2572.58
3,4-dihydroxybenzoic acid	ari	-0.01	(-0.02– 0.01)	0.46	-2572.62
	log	-0.23	(-0.78– 0.32)	0.44	-2572.58
Cinnamoylglycine	ari	0.01	(-0.01– 0.02)	0.44	-3174.17
	log	0.19	(-0.36– 0.74)	0.53	-3174.27
3-Hydroxybenzyl alcohol	ari	-0.01	(-0.02– 0.01)	0.45	-2558.06
	log	-0.15	(-0.71– 0.40)	0.62	-2558.23
3,4-Dihydroxybenzeneacetic acid	ari	-0.01	(-0.02– 0.01)	0.46	-2579.44
	log	-0.15	(-0.70– 0.41)	0.63	-2579.62
Salicylic acid	ari	0.01	(-0.01– 0.02)	0.48	-2435.10
	log	0.07	(-0.49– 0.62)	0.86	-2435.25
Glucuronic acid and/or isomers	ari	0.01	(-0.01– 0.02)	0.49	-2470.30
	log	0.15	(-0.40– 0.70)	0.62	-2470.42
Glycocholic acid isomer 2	ari	-0.01	(-0.02– 0.01)	0.50	-2222.84
	log	0.08	(-0.48– 0.63)	0.83	-2223.29
Gentisic acid	ari	-0.01	(-0.02– 0.01)	0.50	-2785.85
	log	-0.12	(-0.67– 0.44)	0.72	-2786.03

Azelaic acid	ari	-0.01	(-0.02– 0.01)	0.51	-3004.31
	log	-0.15	(-0.70– 0.40)	0.63	-3004.39
Nicotinuric acid isomer	ari	-0.01	(-0.02– 0.01)	0.52	-2571.96
	log	-0.14	(-0.70– 0.41)	0.63	-2572.07
Tetrahydrohippuric acid	ari	0.01	(-0.01– 0.02)	0.52	-2750.30
	log	0.14	(-0.42– 0.69)	0.66	-2750.40
Pyroglutamic acid	ari	0.00	(-0.01– 0.02)	0.53	-2757.24
	log	0.17	(-0.38– 0.72)	0.57	-2757.28
p-Hydroxyhippuric acid	ari	-0.00	(-0.02– 0.01)	0.60	-3146.31
	log	-0.18	(-0.73– 0.37)	0.54	-3146.26
2-Isopropylmalic acid	ari	0.00	(-0.01– 0.02)	0.56	-2946.72
	log	0.10	(-0.45– 0.65)	0.74	-2946.84
Xanthosine	ari	-0.00	(-0.02– 0.01)	0.58	-2689.75
	log	-0.14	(-0.70– 0.41)	0.64	-2689.78
Taurine	ari	-0.00	(-0.02– 0.01)	0.8	-2598.95
	log	-0.16	(-0.71– 0.39)	0.6	-2598.83
3-Methyl-2-oxovaleric acid	ari	-0.00	(-0.02– 0.01)	0.62	-2892.88
	log	-0.13	(-0.68– 0.42)	0.68	-2892.93
3,3-Dimethylglutaric acid	ari	0.00	(-0.01– 0.02)	0.83	-2541.43
	log	0.13	(-0.42– 0.68)	0.68	-2541.36
Sulfosalicylic acid isomer	ari	-0.00	(-0.02– 0.01)	0.70	-3077.88
	log	-0.05	(-0.60– 0.50)	0.89	-3077.96
Ketoleucine	ari	-0.00	(-0.02– 0.01)	0.73	-2732.25
	log	-0.10	(-0.66– 0.45)	0.74	-2732.27
(2-methoxyethoxy)propanoic acid isomer	ari	-0.00	(-0.02– 0.01)	0.79	-2485.21
	log	0.00	(-0.55– 0.55)	1.00	-2485.31
2-Methylhippuric acid	ari	0.00	(-0.01– 0.02)	0.89	-2619.73
	log	0.09	(-0.46– 0.64)	0.79	-2619.70
3-Hydroxyphenylacetic acid	ari	-0.00	(-0.02– 0.01)	0.96	-2839.26
	log	-0.08	(-0.63– 0.47)	0.81	-2839.22
Monoethyl phthalate	ari	0.00	(-0.01– 0.02)	0.82	-2512.30
	log	0.05	(-0.51– 0.60)	0.91	-2512.33
N-Acetylleucine	ari	-0.00	(-0.02– 0.01)	0.82	-2541.55
	log	-0.06	(-0.61– 0.49)	0.88	-2541.57
alpha-N-Phenylacetyl-glutamine	ari	-0.00	(-0.02– 0.01)	0.83	-3396.04
	log	-0.06	(-0.62– 0.49)	0.86	-3396.05
o-methylhippuric acid	ari	0.00	(-0.01– 0.02)	0.83	-2605.05
	log	0.02	(-0.53– 0.57)	0.97	-2605.07
m-methylhippuric acid	ari	0.00	(-0.01– 0.02)	0.83	-2605.05
	log	0.02	(-0.53– 0.57)	0.97	-2605.07
p-methylhippuric acid	ari	0.00	(-0.01– 0.02)	0.83	-2605.05
	log	0.02	(-0.53– 0.57)	0.97	-2605.07
p-Hydroxyphenylacetic acid	ari	-0.00	(-0.02– 0.01)	0.84	-2650.88
	log	-0.03	(-0.58– 0.52)	0.96	-2650.91
Chenodeoxycholic acid isomer	ari	0.00	(-0.01– 0.02)	0.87	-2384.89
	log	0.07	(-0.48– 0.62)	0.84	-2384.88
9-Methylxanthine	ari	-0.00	(-0.02– 0.01)	0.84	-3180.67
	log	-0.06	(-0.61– 0.50)	0.88	-3180.68
Hydroxybenzyl alcohol isomer	ari	0.00	(-0.01– 0.02)	0.89	-2635.57
	log	0.07	(-0.48– 0.62)	0.85	-2635.56

6-(2-hydroxyethoxy)-6-oxohexanoic acid	ari	-0.00	(-0.02– 0.01)	0.86	-2346.24
	log	-0.03	(-0.58– 0.52)	0.95	-2346.26
Malic acid	ari	0.00	(-0.01– 0.02)	0.95	-2555.95
	log	0.06	(-0.49– 0.61)	0.87	-2555.94
Hexanoylglycine	ari	-0.00	(-0.02– 0.01)	0.87	-2710.24
	log	-0.02	(-0.57– 0.53)	0.98	-2710.26
Phenol sulfate	ari	-0.00	(-0.02– 0.01)	0.98	-3421.62
	log	-0.05	(-0.60– 0.50)	0.89	-3421.60
Asp-Leu/Ile isomer 1	ari	-0.00	(-0.01– 0.01)	0.99	-2629.28
	log	0.04	(-0.51– 0.59)	0.92	-2629.29
Methyl (hydroxymethyl)pyrrolidine-carboxylate	ari	0.00	(-0.01– 0.02)	0.96	-2780.32
	log	0.02	(-0.53– 0.57)	0.98	-2780.31
Methyl (hydroxy)piperidine-carboxylate	ari	0.00	(-0.01– 0.02)	0.96	-2780.32
	log	0.02	(-0.53– 0.57)	0.98	-2780.31
Suberic acid	ari	-0.00	(-0.01– 0.01)	0.98	-2811.84
	log	0.03	(-0.52– 0.58)	0.96	-2811.83

Table 1: min p-Values of two models

Under a linear model (i.e., for the metameter "ari") investigate the association between age and all metabolites simultaneously

```
### linear effects only
make_str <- function(a)
  as.mlt(BoxCox(as.formula(paste(a, " ~ ageari")), data = dd))
res <- lapply(analyt, make_str)
names(res) <- analyt
### construct mmm / glht
a1 <- do.call("mmm", res)
a2 <- rep("ageari = 0", length(analyt))
names(a2) <- analyt
a2 <- as.list(a2)
a2 <- do.call("mlf", a2)
m <- glht(a1, a2)
### compute confidence internals
ci <- confint(m)
```

```
ci <- ci$confint
ci.o <- ci[order(ci[,1]),]
ID <- which((ci.o[, 3] <= 0 | 0 <= ci.o[, 2]))
w.n<-rownames(ci.o[ID,])
w.n<-sub(": ageari","",w.n)
par(mar=c(5, 4, 4, 8) + 0.05)
plot(ci.o[,1], 1:nrow(ci.o), pch = 20, main="Metabolites vs. Age",
     xlab="Effects",ylab="Metabolites i=1,...,120")
colors <- rep("orange", nrow(ci.o))
colors[ID] <- "blue"
segments(ci.o[,2], 1:nrow(ci.o), ci.o[,3], 1:nrow(ci.o),col=colors)
abline(v = 0)
axis(4,at=which(colors=="blue"), labels=w.n,las=2,cex.axis=0.25,tck=-0.01)
```

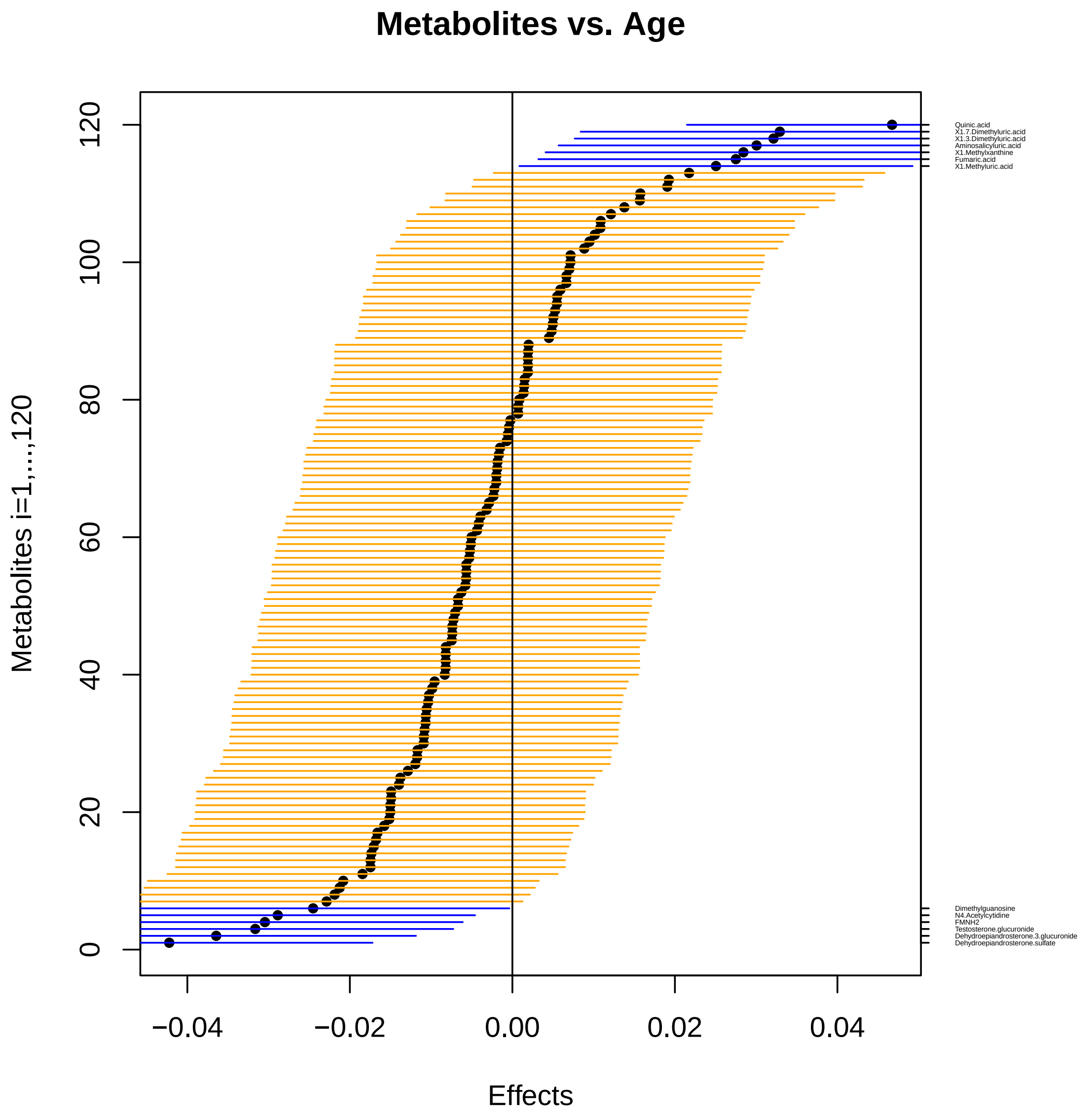

Figure 3: Impact of age on 120 urine metabolites for 183 adults under a linear model, visualized by confidence intervals, ordered by increasing effects, given as horizontal lines around black circles. The blue lines are the metabolites that show association with age.

```
## R version 4.2.2 (2022-10-31 ucrt)
## Platform: x86_64-w64-mingw32/x64 (64-bit)
## Running under: Windows 10 x64 (build 17763)
##
## Matrix products: default
##
## locale:
## [1] LC_COLLATE=German_Germany.1252  LC_CTYPE=German_Germany.1252
## [3] LC_MONETARY=German_Germany.1252 LC_NUMERIC=C
## [5] LC_TIME=German_Germany.1252
##
## attached base packages:
## [1] stats     graphics  grDevices utils     datasets  methods   base
##
## other attached packages:
##  [1] gdata_2.18.0.1    data.table_1.14.8 tibble_3.1.8      lattice_0.20-45
##  [5] ggplot2_3.3.6     xtable_1.8-4      tukeytrend_0.7    multcomp_1.4-20
##  [9] TH.data_1.1-1     MASS_7.3-58.1     survival_3.4-0    mvtnorm_1.1-3
## [13] tram_0.8-0        mlt_1.4-3         basefun_1.1-3     variables_1.1-1
## [17] knitr_1.40
##
## loaded via a namespace (and not attached):
##  [1] Rcpp_1.0.9            tidyr_1.2.0           gtools_3.9.3
##  [4] zoo_1.8-10            digest_0.6.29         utf8_1.2.2
##  [7] R6_2.5.1              backports_1.4.1       alabama_2022.4-1
## [10] evaluate_0.16         highr_0.9             pillar_1.8.1
## [13] rlang_1.0.5           rstudioapi_0.14       minqa_1.2.4
## [16] nloptr_2.0.3          Matrix_1.5-1          labeling_0.4.2
## [19] splines_4.2.2         lme4_1.1-30           polynom_1.4-1
## [22] stringr_1.4.1         munsell_0.5.0         broom_1.0.1
## [25] compiler_4.2.2        numDeriv_2016.8-1.1   xfun_0.32
## [28] pkgconfig_2.0.3       mgcv_1.8-41           tidyselect_1.1.2.9000
## [31] coneproj_1.16         BB_2019.10-1          quadprog_1.5-8
## [34] codetools_0.2-18      fansi_1.0.3           dplyr_1.0.10
## [37] withr_2.5.0           grid_4.2.2            nlme_3.1-160
## [40] gtable_0.3.1          lifecycle_1.0.2       orthopolynom_1.0-6.1
## [43] magrittr_2.0.3        scales_1.2.1          cli_3.3.0
## [46] stringi_1.7.8         farver_2.1.1          generics_0.1.3
## [49] vctrs_0.4.1           boot_1.3-28           sandwich_3.0-2
## [52] Formula_1.2-4         tools_4.2.2           glue_1.6.2
## [55] purrr_0.3.4           pbkrtest_0.5.1        parallel_4.2.2
## [58] colorspace_2.0-3
```
